# The Role of NAD+, SIRTs Interactions in Stimulating and Counteracting Carcinogenesis

**DOI:** 10.3390/ijms24097925

**Published:** 2023-04-27

**Authors:** Ekaterina Podyacheva, Yana Toropova

**Affiliations:** Almazov National Medical Research Centre, Ministry of Health of the Russian Federation, 197341 Saint-Petersburg, Russia; ekaterinapodyachevaspb@gmail.com

**Keywords:** cancer, NAD^+^ metabolism, NAD^+^ precursors, SIRTs, PARPs, Warburg effect

## Abstract

The World Health Organization has identified oncological diseases as one of the most serious health concerns of the current century. Current research on oncogenesis is focused on the molecular mechanisms of energy-biochemical reprogramming in cancer cell metabolism, including processes contributing to the Warburg effect and the pro-oncogenic and anti-oncogenic roles of sirtuins (SIRTs) and poly-(ADP-ribose) polymerases (PARPs). However, a clear understanding of the interaction between NAD^+^, SIRTs in cancer development, as well as their effects on carcinogenesis, has not been established, and literature data vary greatly. This work aims to provide a summary and structure of the available information on NAD^+^, SIRTs interactions in both stimulating and countering carcinogenesis, and to discuss potential approaches for pharmacological modulation of these interactions to achieve an anticancer effect.

## 1. Introduction

Cancer is one of the leading causes of death worldwide. Maintenance of replicative immortality, evasion of growth suppressors, induction of angiogenesis, activation of invasion and metastasis, reprogramming of energy metabolism, and evasion of the immune system contribute to the uncontrolled and abnormal growth of cancer cells that are difficult to treat [1,2].

In oncogenetic investigations, the molecular mechanisms of energy-biochemical reprogramming of cancer cell metabolism are being actively discussed. The article delves into the mechanisms that disrupt the oxidative phosphorylation systems, resulting in the activation of anaerobic glycolysis. This leads to the production of significant quantities of lactate, nicotinamide adenine dinucleotide (NAD^+^) [3], glyceraldehyde phosphate dehydrogenase [4], and increased pentose phosphate and serine pathways. The article also investigates how these processes affect the levels of NADPH and reactive oxygen species [5]. Moreover, both pro-oncogenic and anti-oncogenic roles of sirtuins (Silent Information Regulator 2 proteins, SIRTs) and poly-(ADP-ribose) polymerases (PARPs) are known today, which are actively involved in the regulation of cell energy metabolism together with NAD^+^.

However, there are several issues that have not been explored yet, including the impact of changes in NAD^+^/NADH balance on the functioning of SIRTs and PARPs, the molecular mechanisms that regulate the movement of sirtuins between cell compartments during cancer development [6,7], the multidirectional differences in SIRTs expression within the same type of cancer process [8,9,10], the molecular mechanisms underlying interactions between all classes of sirtuins during cancer development and beyond, and the effect of these interactions on the functioning of PARPs and NAD^+^ [11,12]. Additionally, the impact of these signaling pathways on the formation of specific signs of cancer growth remains to be fully understood. In addition, the role of both inhibitors and activators of sirtuins is widely discussed, which, as a result, have a dual effect on both the cancer and healthy tissues [13].

An unequivocal comprehension of the interactions among NAD^+^, SIRTs during the development of cancer and their impact on carcinogenesis has not been established yet. Therefore, this review aims to consolidate the existing knowledge on the contribution of NAD^+^, SIRTs interactions in the promotion and inhibition of carcinogenesis. Additionally, potential methods of pharmacological intervention to modulate these interactions and exert an anti-cancer effect are also considered.

## 2. Role of NAD^+^ in Cancer Metabolism

### 2.1. Warburg Effect

**Figure 1:**
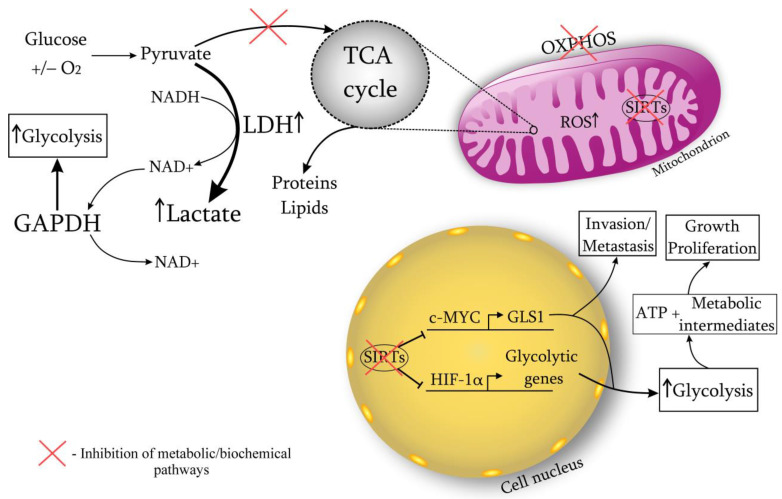
The main mechanisms for the implementation of the Warburg effect. The tumor process disrupts enzymatic systems of oxidative phosphorylation, impairs pyruvate transport into mitochondria, and causes multiple defects in the Krebs cycle. As a result, anaerobic glycolysis becomes the dominant pathway, leading to active synthesis of lactic acid and the development of acidosis in tumor tissue. This disrupts the function of lymphocytes. Increased glucose metabolism also leads to constant activity of LDH, which induces the regeneration of NAD^+^ and affects the concentration of reducing equivalents in mitochondria. The antiapoptotic protein GAPDH, which is necessary for the function of NAD^+^, promotes further tumor growth. The excess carbon and reduced equivalents resulting from enhanced glucose metabolism are used for the synthesis of nucleotides, lipids, and proteins. Research has shown that sirtuins can suppress the expression of HIF-1α, thus inhibiting glycolysis by blocking the expression of glycolytic enzymes. SIRT6 also suppresses cMYC and its targets, such as GLS1, to inhibit glutamine catabolism, which stimulates enhanced glucose uptake by tumor tissue. DNA damage in the nucleus induces the expression of SIRT4, which inhibits the activity of glutamate dehydrogenase, thereby regulating glutamine synthesis. ATP, adenosine triphosphate; GAPDH, glyceraldehyde phosphate dehydrogenase, LDH, lactate dehydrogenase; OXPHOS, oxidative phosphorylation; ROS, reactive oxygen species; TCA cycle, citric acid cycle.

A cancer cell is characterized by a metabolic restructuring, i.e., biochemical atypism (Warburg effect) (Figure 1). Even in the presence of oxygen, oxidative phosphorylation is dominated by anaerobic glycolysis, which leads to the lactate accumulation and the medium acidification to pH 7.2 (normally 7.4). The development of cancer involves a disruption in the enzymatic systems responsible for oxidative phosphorylation. Specifically, the number of functioning mitochondria is reduced by 20–50%, the expression of the inhibitory subunit of ATP synthase is affected, and there are disturbances in the transport of pyruvate into mitochondria. Additionally, multiple defects can arise in the Krebs cycle, such as a defect in the gene responsible for isocitrate dehydrogenase.

There is a competition for availability of glucose between cancer cells and cancer-infiltrating lymphocytes in favor of the former [14,15,16] due to the developing acidosis [17]. The dysregulation of glucose homeostasis leads to impaired functioning of lymphocytes. Anaerobic glycolysis is faster than oxidative phosphorylation but lower in quality. Glucose is converted to lactate through anaerobic glycolysis at a rate 10–100 times faster than glucose oxidation through mitochondria. Research suggests that the ATP needed for cell growth and division is much less compared to the amount needed for maintaining cell viability [18]. Presumably the excess carbon, formed due to increased glucose metabolism, used for the synthesis of nucleotides, lipids, proteins, and other building blocks. Actively proliferating cells require reducing equivalents in the form of NADPH more than ATP [19,20]. This hypothesis is confirmed by the fact of high glutamine consumption by cancer cells [21]. Glutamine metabolism provides cancer cells with the necessary substrates to maintain high proliferative activity, controls the redox potential through the NADPH regulation. Elevated levels of glutamine promote increased uptake of glucose by the cancer, and it can also be converted to lactate [22]. The metabolism of glucose and glutamine are closely related to each other.

When the rate of glycolysis fluctuates, cellular mechanisms begin to actively maintain redox homeostasis. NADH plays the key role in these processes, which is available in mitochondria for electron transport. The malate-aspartate shuttle through the mitochondria is able to restore the NADH imbalance to a certain level of glycolysis. However, when the rate of glucose metabolism increases, there is a switch to lactate dehydrogenase (LDH) and the conversion of pyruvate to lactate, which promotes NAD^+^ regeneration [22]. Therefore, LDH is essential for the viability of cancer cells. Research confirms cancer cell dependence on LDH due to its role in NAD^+^ regeneration [15,23]. In vivo studies on mice have demonstrated that by targeting LDH, it is possible to lower NAD^+^ levels and delay the onset and spread of cancers. Coordinated modulation of both LDH and NAD^+^ has been observed to enhance cancer cell mortality, suggesting that reduced NAD^+^ availability is responsible for cancer cell demise [14,15]. Recent work has shown that LDHA activates the small GTPase Rac1, contributing to the development of cancer, regardless of the activity of its glycolytic enzyme [24]. The process of converting pyruvate to lactate via LDH can alter the homeostasis of ROS generation by affecting the concentration of reducing equivalents in mitochondria. Additionally, with the Warburg effect, other metabolic pathways are enhanced, such as the pentose phosphate and serine pathways, which produce NADPH, modulating ROS levels [5]. Moreover, NAD^+^ is required for the functioning of the enzyme glyceraldehyde phosphate dehydrogenase (GAPDH). This glycolytic enzyme is responsible for the conversion of glyceraldehyde-3-phosphate to 1,3-biphosphoglycerate, catalyzing the sixth step of glycolysis [25]. It has been shown that GAPDH, having glycolytic and anti-apoptotic functions, promotes the proliferation and protection of cancer cells. GAPDH is overexpressed in multiple human cancers and its expression is positively correlated with cancer progression [4,26]. Experiments have shown that when NAD^+^ is depleted (in addition to reducing LDH), GAPDH activity also decreases. In this regard, there is a blockage of glycolysis, aging of cancer cells [27,28,29,30] (Figure 1).

Moreover, it has been suggested that glycolytic metabolism affects chromatin structure [31]. Wellen et al. wrote that elevated levels of acetyl-CoA may be sufficient to bring cells into the growth phase via histone acetylation [32]. The glucose removal results in the loss of acetylation on several histones and causes a decrease in the genes transcription involved in glucose metabolism. Thus, acetyl-CoA, a substrate for histone acetylation, can be regulated by glucose levels [33,34].

Therefore, the main biochemical event characterizing the cancer process is the restructuring of metabolism from oxidative phosphorylation to anaerobic glycolysis, which is more favorable in terms of speed (Warburg effect). These changes are necessary for cancer cells to maintain the high NAD^+^ production required to support numerous cellular processes during cancer growth. In addition, an increase in lactate levels contributes to the acidification of the cancer environment, which provides some protective function against the body’s immune system, promotes further invasion and metastasis.

### 2.2. NAD^+^ Metabolism

**Figure 2:**
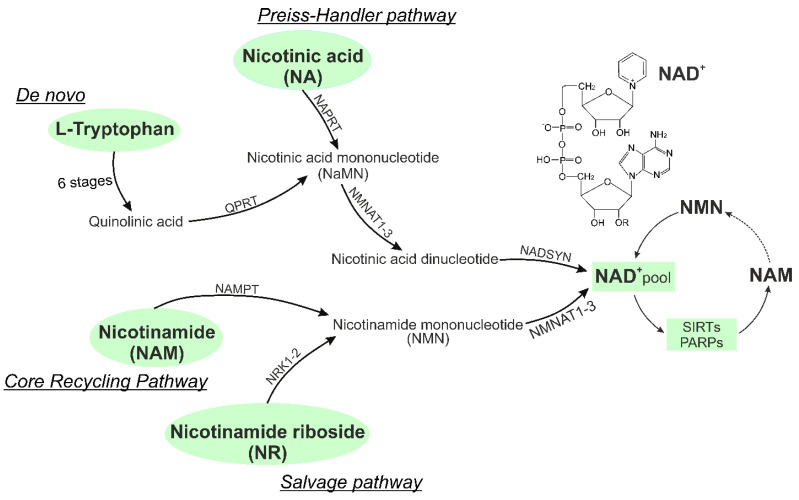
NAD^+^ synthesis pathways. In the De novo biosynthetic pathway, tryptophan is converted to quinolinic acid in 6 steps of enzymatic reactions, which is then converted to nicotinic acid mononucleotide (NAMN) by the enzyme quinolinate phosphoribosyltransferase (QPRT). The Preiss-Handler pathway is initiated by NA phosphoribosyltransferase (NAPRT) to form NAMN. NAMN is further converted to NA adenine dinucleotide (NAAD) by NMN adenylyl transferase enzymes (NMNAT1-3). Finally, NAAD is converted to NAD^+^ via an amidation reaction catalyzed by the enzyme NAD^+^ synthase (NADSYN). The Core Recycling and Salvage Pathways are the shortest pathways for NAD^+^ synthesis from NAM and NR (2 steps). NAM is converted to NMN by the rate-limiting nicotinamide phosphoribosyl transferase (NAMPT). NMN is also a product of NR phosphorylation by NR kinases (NRK1-2). The subsequent conversion of NMN to NAD^+^ is catalyzed by NMNAT1-3 enzymes. Further, the synthesized NAD^+^ is used in the functioning of NAD^+^-dependent enzymes (SIRTs, PARPs). As a result of their reaction (deacetylation, poly-ADP-ribosylation, respectively), NAM is formed again, which later turns into NMN using the intracellular form of NAM-phosphoribosyltransferase. NA, nicotinic acid; NAAD, NA adenine dinucleotide; NADSYN, NAD+ synthetase; NAM, nicotinamide; NAMN, NA mononucleotide; NMN, NAM mononucleotide; NMNAT, NMN adenylyltransferase; NR, nicotinamide riboside; NRK, NR kinase.

Four pathways have been identified for the biosynthesis of NAD^+^ (as shown in Figure 2): (1) *the De novo pathway* (also known as *the kynurenine pathway*), which involves eight steps and metabolizes tryptophan; (2) *the Preiss-Handler pathway*, which uses both exogenous and endogenous nicotinic acid (NA); (3) *the Salvage pathway*, which produces NAD^+^ from nicotinamide riboside (NR); and (4) *the Core Recycling pathway*, which recycles NAD^+^ from nicotinamide (NAM) [35]. In each of these pathways its own enzyme systems are uses. In *the De novo pathway*, after 6 steps of enzymatic reactions, quinolinic acid (QA) is formed from tryptophan, which is then converted into nicotinic acid mononucleotide (NAMN) by the enzyme quinolinate phosphoribosyltransferase (QPRT). NAMN is also synthesized in *the Preiss-Handler pathway* by transferring the phosphoribosyl part from phosphoribosyl pyrophosphate (PRPP) to nicotinic acid (NA) by NA phosphoribosyltransferase (NAPRT). Moreover, PRPP can be considered as a linking molecule between glucose metabolism and NAD^+^ biosynthesis, since PRPP is the central product of the pentose phosphate pathway (PPP) and the source of the NAD^+^ sugar moiety. NAM in *the Core Recycling pathway* is converted to nicotinamide mononucleotide (NMN) by the rate-limiting enzyme nicotinamide phosphoribosyl transferase (NAMPT), whereas NMN is also a product of NR phosphorylation by NR kinases (NRK1-2).

The subsequent conversion of NAMN and NMN to NAD^+^ is catalyzed by the respective enzymes’ nicotinamide/nicotinic acid mononucleotide adenylyltransferases (NMNAT1-3).

### 2.3. Pro-Oncogenic Effects of NAD^+^

Cancers are classified into two categories depending on the predominant pathway of NAD^+^ synthesis: NAPRT- and NAMPT-dependent cancer. Tissues highly expressing NAPRT become dependent on NAPRT for survival after malignant transformation. On the other hand, NAD^+^ supply of cancer that lack NAPRT expression is primarily dependent on NAMPT. Chowdhry et al. demonstrated that overexpression of NAPRT in a NAMPT-dependent cancer (and vice versa) does not change either the NAD^+^ level or its metabolic pathway. Moreover, in NAPRT- and NAMPT-dependent cancers where the related enzyme is deficient, an alternative route for producing NAD^+^ via NRK1 is activated [36]. Dual inhibition of NRK1 and NAMPT result in more effective cancer suppression in vivo through inhibition of NAD^+^ [36].

High expression of NAMPT is characteristic of a huge range of solid and hematological NAMPT-dependent cancer, e.g., cancer of the stomach, pancreas, prostate, breast, ovary, melanoma, lymphoma, sarcoma, glioma, thyroid carcinoma [37]. Both intracellular (iNAMPT) and extracellular (eNAMPT) forms of NAMPT have pro-oncogenic and pro-inflammatory effects [38,39], and their increased expression is associated with a poor prognosis in cancer. eNAMPT is involved in modulating the cancer microenvironment by enhancing its metabolism [39], activates the pro-inflammatory NF-κB pathway by binding to the Toll-like receptor 4 (TLR4) [40].

To date, such molecular pathways are known that regulate the expression and activity of NAMPT in cancers, such as c-MYC/NAMPT/SIRT1, HMGA1/NAMPT/NAD^+^, FOXO1/NAMPT, NAMPT/miRNA, SIRT6/SIRT1/NAMPT, C/EBPβ/NAMPT (Table 1). Mensen et al. described the c-MYC/NAMPT/SIRT1 positive feedback loop [41,42]. c-MYC, interacting with NAMPT promoter, induces its expression. This leads to an increase NAD^+^ levels and to SIRT1 activation. SIRT1 is able to stabilize and enhance the transcriptional activity of c-MYC, as well as to weaken p53 activity and inhibit c-MYC-induced apoptosis, thereby stimulating cancer growth [41,42,43]. The high-mobility group A (HMGA1) protein participates in the regulation of NAMPT expression through NAD^+^-mediated enhancement of the NF-κB activity, contributing to the inflammatory environment and stimulating cancer progression [44]. Forkhead box O1 (FOXO1) is known as a cancer suppressor and it is able to suppress the NAMPT expression [45]. The insulin–PI3K–AKT signaling pathway produces a response in counteraction to FOXO1. Studies conducted between 2018 and 2020 have shown that elevated levels of certain microRNAs (miR-381, miR-206, miR-494, miR-154, miR-23b, miR-26b) have a negative impact on the expression of NAMPT in breast cancer [46,47,48], pancreatic cancer [49], melanoma [50], and colorectal cancer [51]. SIRT6 and SIRT1 enhance the enzymatic NADPH activity by direct deacetylation of the protein, protecting cancer cells from oxidative stress [52]. Mesenchymal glioblastoma stem cells enhance NAMPT expression via transcription factor C/EBPβ [53]. Currently, NAPRT-dependent cancers are less well-known, but such mechanisms that regulate NAPRT gene expression as alternative splicing, promoter hypermethylation, mutations in transcription factor binding sites are described in Duarte-Pereira et al. [54].

### 2.4. NAMPT Inhibitors

There are specific and double NAMPT inhibitors that can be distinguished (Table 2). The specific ones include FK866 (APO866/WK175), CHS-828 (GMX1778), GMX1777 (EB1627), OT-82. Leo Pharma AS developed an anticancer drug called CHS-828, which contains pyridyl cyanoguanidine. This drug showed strong anticancer properties against breast and lung cancer cell lines, and it achieved this by inhibiting NAMPT and depleting NAD^+^ [55,56]. The FK866 is a competitive inhibitor of NAMPT according to crystallographic studies [57]. The FK866 has shown reliable anticancer efficacy in preclinical studies in the treatment of a wide range of solid and hematological cancers both in vitro and in vivo [58,59,60]. Moreover, studies were conducted in which it was shown that the cytotoxicity of the FK866 increased under conditions of energy starvation (low glucose levels). Pharmaceutical inhibition of lactate dehydrogenase indirectly increased the effect of the FK866 in glioma cells, Burkitt lymphoma [29,61]. It is also known that the MAP-kinase pathway mediates cancer cell proliferation, angiogenesis, and metastasis. The use of MAP-kinase inhibitors simultaneously increased FK866-mediated cell death [62]. In addition, the combination of NAMPT and CD73/38 inhibition synergistically reduced intra-cancer levels of NAD^+^, NMN, ATP, decreased the proliferation of ovarian carcinoma cells in vivo and improved the survival of animals with cancers [63]. This drug is still widely used in the study of oncological diseases. The GMX1777 was created as a prodrug to improve the pharmacokinetics and solubility profile of the CHS-828 [64]. The OT-82 is a novel NAMPT-inhibitor with subsequent depletion of NAD^+^ and ATP, and also causes apoptotic cell death. This drug demonstrated its stronger activity against hematopoietic malignant neoplasms [65].

Dual NAMPT inhibitors include such agents as KPT-9274 (ATG-019), STF-31, and Chidamide (Table 2). The viability, invasion, and migration of cancer cells are reduced by the KPT-9274, which is a hybrid inhibitor of NAMPT and serine/threonine–p21-activated kinase 4 (PAK4). Additionally, it induces apoptosis, leads to NAD^+^ depletion, and decreases the activity of SIRT1 [66]. The chidamide is a dual histone deacetylase (HDAC)/NAMPT inhibitor that is used to treat cutaneous T-cell lymphoma. The STF-31 is a hybrid inhibitor of NADPH and glucose transporter 1 (GLUT 1) [67].

**Table 2:** The main molecular effects of NAMPT-inhibitors.

NAMPT Inhibitors					
Specific			Dual		
Name	References	Target Molecules	Name	References	Target Molecules
CHS-828 (GMX1778)	[55,56]	Anticancer activity in lung and breast cancer cell lines; causes NAD^+^ depletion	Chidamide (HBI-8000)	[67]	HDAC/NAMPT inhibitor; suppresses cyclin E1; causes NAD^+^ depletion and apoptosis
FK866 (APO866/WK175)	[57,58,59,60]	Anticancer activity in the treatment of a wide range of solid and hematological cancers; causes NAD^+^ depletion and apoptosis	STF-31	[67]	NAMPT and GLUT1 inhibitor; impairs glucose uptake, lowers NAD^+^ levels and causes apoptosis
GMX1777 (EB1627)	[64]	Improved version of CHS-828	KPT-9274 (ATG-019)	[66]	NADPH and PAK4 inhibitor; reduces viability, invasion/migration of cancer cells and induces apoptosis; suppresses c-MYC and cyclin D1; causes depletion of NAD^+^ and SIRT1
OT-82	[65]	Strong anticancer activity against hematopoietic malignancies; causes depletion of NAD^+^, ATP and apoptosis			

It is important to note that the NAMPT inhibition affect on the functioning of mitochondria. Several studies have demonstrated that NAMPT inhibition causes mitochondrial dysfunction in cancer cells in breast carcinoma, leukemia, pancreatic cancer and Burkitt’s lymphoma [62,68,69]. However, there is evidence suggesting that cancer cell lines with impaired citric acid cycle are vulnerable to NAD^+^ depletion through the use of FK866 and GMX1778. This means that cells with properly functioning mitochondria can withstand inhibition of NAMPT, whereas those with faulty mitochondria are more prone to cyto-toxicity [30,70]. In addition, with the mitochondrial dysfunction development, the balance of ROS generation is disrupted. Cancer cells, like healthy ones, must constantly maintain an antioxidant defense system in order to survive. Studies conducted on various cancer models, such as colon cancer, prostate cancer, breast cancer, pancreatic cancer, and leukemia, have demonstrated that NAD^+^ plays a crucial role in regulating oxidative stress. Knocking down NAMPT, an enzyme involved in NAD^+^ biosynthesis, has been found to lead to an increase in the production of ROS [68,71,72,73]. This is attributed to a reduction in the levels of key antioxidant defense proteins, including catalase mRNA, superoxide dismutase, and glutathione [71,74,75]. Thus, NAD^+^ depletion makes cancer cells sensitive to oxidative damage.

Besides the resilience of cells containing viable and fully operational mitochondria to NAMPT inhibitors, it is crucial to consider the potential severe adverse effects on healthy tissues. Von Heideman et al.’s research in phase I clinical trials revealed notable toxicity from NAD^+^ inhibitors [13].

Consequently, combining NAD^+^ inhibitors with treatments targeting energy production sources such as glycolysis and glutamine can enhance cancer cell death and open up new therapeutic avenues.

## 3. SIRTs/PARPs as Pro- and Anti-Oncogenic Targets

### 3.1. Sirtuins

Sirtuins have garnered significant attention for their involvement in cancer development and their potential as targets for new therapeutic interventions. This is because sirtuins play a crucial role in regulating metabolic reprogramming, modifying the cancer microenvironment, and maintaining genome stability [76,77,78,79]. Sirtuins are a family of evolutionarily conservative NAD^+^-dependent proteins, the third class of histone deacetylases with deacetylase or ADP-ribosyltransferase activity. This family is divided into 7 classes (SIRT1-7), which is encoded in the human genome, and each of these classes is located and performs its functions in certain cellular compartments (SIRT1,2,6,7—in the nucleus, SIRT1,2—in the cytoplasm, SIRT3,4,5—in mitochondria) (Table 3).

#### 3.1.1. SIRT1

SIRT1 transcription regulation is influenced by p53, E2F1, and the hypermethylated in cancer 1 protein (HIC1). The protein p53 can inhibit SIRT1 transcription by binding to specific promoter elements, which creates a negative feedback loop. Following DNA damage, SIRT1 deacetylation of p53 prevents its activation through a p53-dependent pathway [80]. The acetylation status of p53 plays a crucial role in its function as a transcription factor by enabling its capacity to inhibit cell proliferation and trigger programmed cell death. Thus, this interaction is essential for the proper functioning of p53 [81,82]. Transcription factor E2F1 also actions on the principle of negative feedback with SIRT1. Its role is to activate several apoptotic genes (TP53, TP73 and APAF1) to implement apoptosis for DNA damage through both p53-dependent and p53-independent mechanisms [83]. The E2F1 is a substrate of SIRT1, and E2F1 deacetylation inhibits its activity as a transcription activator [83]. Thus, SIRT1 through the E2F1 regulation can determine the apoptotic fate of the cells. HIC1 is a tumor suppressor gene, and the loss of its function contributes to oncogenesis by increasing the level of SIRT1 expression [84]. HIC1 and C-terminal-binding protein 1 (CTBP1) form a complex and bind to the enhancer elements of the SIRT1 promoter, resulting in the inhibition of SIRT1 expression. This inhibition allows for the acetylation of p53 [85]. SIRT1′s carcinogenic impact arises from its ability to deactivate p53 by increasing its deacetylation, which allows for the avoidance of p53-mediated apoptosis. Thus, SIRT1 promotes cell survival following DNA damage [10]. HUR and miR-34a are oncosuppressors that participate in the regulation of SIRT1 mRNA. They achieve this by binding to the 3′-UTR of SIRT1 mRNA. HUR assists in stabilizing the SIRT1 transcript. However, in the event of DNA damage, the interaction between HUR and SIRT1 may cause a decrease in SIRT1 levels, which ultimately leads to p53-mediated apoptosis [86]. miR-22, miR-93, miR-217 and miR-449 also suppress SIRT1 to control cancer genesis by inhibiting cell proliferation and stimulating cellular aging [87].

The mechanisms of SIRT1 regulation are still being investigated, especially in aspects of its participation in the development or suppression of oncological diseases. SIRT1 has a repressive effect on the tumor suppressor p53, KU70, NF-κB, PGC-1α and members of the forkhead box (FOXO) transcription factor family [88,89]. Deacetylation of FOXO proteins (specifically, FOXO1, FOXO3, and FOXO4) by SIRT1 in response to double-stranded DNA breaks has been shown to trigger cell cycle arrest and enhance the organism’s resistance to oxidative stress. Moreover, this process can also reduce FOXO-mediated apoptosis, resulting in an extension of the organism’s lifespan [90]. This path is similar to the operation of E2F1/SIRT1 signaling pathway. Knockdown or inhibition of NAMPT contributes to a decrease in FOXO3a levels and increases the inhibitory FOXO3a acetylation in prostate cancer cells. The SIRT1-dependent glycolytic stimulating gene c-MYC requires NAMPT function for SIRT1-mediated activation [41]. Increased pro-oncogenic c-Myc can regulate SIRT1 activity, leading to inhibition of pro-apoptotic proteins and stimulation of cell proliferation [41]. Several studies have shown that SIRT1 activation by resveratrol restricts cell growth and reduces cancer formation in Breast cancer type 1 susceptibility protein (BRCA1) deficient cancer cells, as well as in Trp53+/−; Sirt1+ mice/− [91]. Zheng and his colleagues demonstrated that metformin activates the AMPK/SIRT1/NF-κB pathway, leading to the induction of cell pyroptosis, and thus exerting an anti-cancer effect. This pathway promotes the build-up of Bax and the release of cytochrome c, ultimately resulting in the activation of caspase 3 and the cleavage of gasdermin E (GSDME) in cancer cells [92]. Moreover, metformin promotes mitochondrial dysfunction by activating the AMPK/SIRT1 pathway, which contributes to the pyroptosis development. Yang et al., in turn, identified CSAG2 as a novel SIRT1 activator specific for colon, lung and breast cancer, which stimulates the enzymatic activity of SIRT1 through direct binding [93]. SIRT1 also interacts with the Cullin 4B (CUL4B)-Ring E3 (CRL4B) ligase complex, which is responsible for the monoubiquitination of H2AK119 (H2AK119ub1) [94]. Genome-wide analysis of SIRT1/CUL4B targets revealed a cohort of genes, including GRHL3 and FOXO3, critically involved in cell differentiation, growth, and migration [94]. Leng et al. found that SIRT1 together with CUL4B promote proliferation, autophagy, and invasion of pancreatic cancer cells [94]. Jin et al. noted that breast cancer cells modulate the SIRT1/β-catenin signaling pathways resistant to doxorubicin [95,96]. These pathways effectively inhibit apoptosis and promote cell growth and repair of DNA damage. Simultaneously, according to a study from 2019, the activation of STAT3/MMP-13 signaling that leads to the progression of gastric cancer is caused by a reduction in SIRT1 levels [97]. In addition, deacetylated NF-κB can increase the sensitivity of cells to TNFα-induced apoptosis [98]. SIRT1 is also capable of deacetylating PTEN, a PI3K signaling inhibitor [99]. PI3K/AKT is the most important signaling pathway regulating the cell cycle, respectively, and cancer growth. When PTEN is absent, it strongly inhibits apoptosis and promotes cell proliferation and survival. However, SIRT1 can act as a tumor suppressor by activating PTEN. On the other hand, PI3K activation causes SIRT1 to move into the nucleus, where it can act as a tumor promoter [6,99].

The SIRT1 model of action as an oncosuppressor is not classical. After DNA damage, SIRT1 causes cell growth to stop, not apoptosis [10]. The stimulation of the DNA repair program suggests that the physiological role of SIRT1 in mammalian cells is to prevent oncogenesis and ensure cellular longevity. However, continuous prevention of apoptosis can, on the contrary, contribute to the development of malignant transformation in cells [10]. According to the literature, the relationship between SIRT1 and p53 in cancer depends on their respective presence or absence. SIRT1 facilitates cellular aging and restricts cell proliferation in cells that still possess p53 activity, whereas in cells that lack p53 or HIC1 (which can directly suppress SIRT1 expression), SIRT1 enzyme activity is augmented. In experiments conducted on mice, SIRT1 knockout (SIRT1-KO) animals did not exhibit oncogenesis, but instead displayed hyperacetylated p53, along with heightened apoptosis and genomic disorders [100]. This fact testifies to the cohesive and complex functions of SIRT1 and p53 in order to maintain the stability and integrity of the genome.

Vascularization plays a very important role for the existence and development of cancer tissue. SIRT1 promotes angiogenesis, partly through the gene’s modulation under its control FOXO1, PGC-1α, HIF [101]. The role of SIRT1 in angiogenesis was revealed as a fine-tuning of the Notch signaling pathway. Notch signaling occurs after cleavage of the Notch transmembrane receptor on the “Notch intracellular domain” (NICD) protein, capable of modulating gene expression [23,102]. It is also able to inhibit angiogenesis by suppressing vascular endothelial growth factor (VEGF) signaling. SIRT1 deacetylating NICD participates in inhibition of Notch signal transmission in vivo (Table 4 and Table 5).

#### 3.1.2. SIRT2

SIRT2 expression may vary significantly in various types of cancers, much like SIRT1 [77,103]. As a tumor suppressor, SIRT2 can restrain the expansion of cancer cells by hindering fibroblast activity and cancer angiogenesis. SIRT2 may inhibit cancer cells from producing VEGF, CTGF, and ATP citrate lyase (ACLY) [104]. On the other hand, SIRT2 can also affect the invasion and metastasis of cancer by inhibiting cancer-related macrophages, myeloid cells, and neutrophils, weakening the immune response and helping cancer cells to escape immune surveillance [105]. In addition, SIRT2 participates in the deacetylation of LDH, increases its activity, thereby contributing to the accumulation of lactic acid and proliferation of cancer cells [106].

To date, SIRT2 deacetylating participates in the regulation of p53, FOXO1,3, α-tublin, β-catenin (Akt/GSK-3β/β-catenin), PEPCK1 (RAS/ERK/JNK/MMP-9), and p38-MAPK [107,108]. SIRT2 is also capable of altering APC/C acetylation levels, and using this mechanism, SIRT2 knocks out some of the cancer formations in mice [109]. SIRT2 inhibition can have an impact on the NF-κB/miR-21 pathway, leading to the suppression of cancer cell proliferation. Furthermore, it can raise the levels of caspase 3 and Bax proteins, promoting apoptosis [110]. Bajpe et al. showed that SIRT2 deficiency can lead to cancer cell resistance to drugs acting on the RTK-RAS/RAF-MEK-ERK pathway [111]. Wang et al. revealed that SIRT2-dependent deacetylation of isocitrate dehydrogenase 1 (IDH1) is involved in regulating cellular metabolism and inhibiting metastasis of colorectal cancer in the liver [112]. Colorectal cancer cell research conducted in 2021 revealed that SIRT2 serves as a direct target gene in the Wnt/β-catenin signaling pathway [113]. By inhibiting Wnt/β-catenin signaling, the SIRT2 promoter is activated, leading to enhanced differentiation of cancer cells and the reverse is also true. A recent study showed that the molecular mechanism of LINC00152, underlying the malignant phenotype of gastric cancer cells, is mediated by the miR-138/SIRT2 signaling pathway [114] (Table 4 and Table 5).

#### 3.1.3. SIRT3

High expression of SIRT3 is associated with an unfavorable clinical prognosis of diseases such as esophageal cancer, colon cancer, stomach cancer [115,116,117], third degree breast cancer [118], oral squamous cell carcinoma [119], melanoma [120], kidney cancer [121], and thyroid cancer [122]. Reduced expression of SIRT3 was found to be associated with decreased severity of oncogenesis, as well as reductions in proliferation, colony formation, and cell migration. Furthermore, SIRT3 knockdown increased the effectiveness of cisplatin and tamoxifen treatment for diseases. However, at the same time, numerous studies have shown that the SIRT3 expression also correlates with a good outcome in the treatment of many types of cancer. For instance, breast cancer cells display noticeably lower levels of SIRT3 expression in comparison to normal epithelium. Moreover, decreased SIRT3 expression has been linked to shorter locoregional survival without relapse in breast cancer [123]. SIRT3 can control the distribution of estrogen receptors and the level of oxidative stress, induce glycolysis inhibition. SIRT3 expression is noticeably reduced in cells afflicted with hepatocellular and pancreatic cancers, while a rise in SIRT3 levels corresponds with a heightened chance of overall survival without relapse. This highlights the importance of SIRT3 in the development of these types of cancers [124,125]. Moreover, SIRT3 overexpression inhibits the growth and induces apoptosis of cancer cells, increases their sensitivity to chemotherapeutic agents [126,127]. SIRT3 overexpression has been shown to reduce proliferation and decrease the Warburg-like phenotype of SIRT3-deficient B-cell lymphocytes, whereas knockdown of sirtuin can enhance the migration and invasion of ovarian cancer cells [128,129].

SIRT3 participates in reprogramming of cancer cell metabolism by activating the ROS/HIF-1α pathway: SIRT3-mediated decrease in ROS level induces activation of oxygen-dependent prolyl hydroxylases (PHD) with subsequent degradation of hypoxia-induced factor 1-α (HIF-1α) [130,131]. Furthermore, SIRT3 has the ability to inhibit glycolysis by directly targeting metabolic enzymes. For instance, SIRT3 can repress the activity of HK2 [132] and deacetylate PDC [133] in gastric and breast cancers. Additionally, SIRT3 can deacetylate and impede the activity of GOT2 in pancreatic cells [132]. Thus, SIRT3 potentially contributes to the regulation of the Warburg effect. However, at the same time, SIRT3 can also deacetylate and activate LDH, thereby contributing to anaerobic glycolysis and carcinogenesis [116].

SIRT3, like other sirtuins, has a deacetylating effect on such biological targets as p53, Ku70, FOXO3a, p300/H3-K56, hydroxylmethylglutaryl-CoA synthase 2, glutamate dehydrogenase [134,135,136]. SIRT3 regulates cell proliferation by modulating classical mitogen-activated protein kinases/kinases (MAPK/ERK) and protein kinase B signaling pathways (Akt/PKB). Zhang and Quan et al. revealed the ability of SIRT3 to reduce phosphorylation of ERK1/2 and Akt, thereby causing degradation of the oncoprotein c-MYC, responsible for inhibiting cell proliferation via the SIRT3/Akt signaling pathway [137,138]. Wang et al. demonstrated that SIRT3 can suppress mitosis by deacetylating S-phase kinase-associated protein 2 (Skp2) and counteracting p300-mediated activation of Skp2 [139]. SIRT3 overexpression is also involved in inhibiting pancreatic cancer cell growth by inhibiting iron-regulatory protein 1 (IRP1) enrichment, thereby suppressing transferrin receptor 1 (TfR1) and TfR1-mediated iron uptake and cell growth [140]. According to Dessai et al., aggressive prostate cancer is facilitated by the increased activity of mitochondrial aconitase (ACO2), which results from the repression of mitochondrial SIRT3 transcription by the androgen receptor and its co-regulator steroid receptor coactivator-2 (SRC-2) [141]. Liu and his co-authors demonstrated the oncosuppressive function of SIRT3 by inhibiting AKT-dependent mitochondrial metabolism and epithelial-mesenchymal markers, which leads to ferroptosis and tumor suppression [142]. Inhibiting lung cancer cells is one of the key roles played by melatonin (MLT), as demonstrated by a study conducted in 2012 [143]. The main mechanism of MLT is associated with reprogramming the metabolism of cancer cells, accompanied by a transition from aerobic/anaerobic glycolysis to oxidative phosphorylation. As a result of these changes, MLT can reverse the Warburg effect by increasing the activity of pyruvate dehydrogenase complex when SIRT3 is stimulated [143]. It was discovered through research into the impact of miR-224 on the growth of non-small cell lung cancer induced by cancer-associated fibroblast (NSCLC) that overexpression of SIRT3 can suppress miR-224 by activating AMPK and deactivating mTOR/HIF-1α [144]. The miR-224/SIRT3/AMPK/mTOR/HIF-1α axis forms a positive feedback loop in the modulation of carcinogenic effects in NSCLC. SIRT3 overexpression also inhibited cancer proliferation and promoted apoptosis in the progression of gastric cancer in the study of Ma et al., whereas Notch-1 overexpression reversed the inhibitory effect of SIRT3 [145] (Table 4 and Table 5).

#### 3.1.4. SIRT4

Just like SIRT3, SIRT4 also plays a role in controlling the Warburg effect. Specifically, it hinders glutamine metabolism by ADP-ribosylating GDH, which restricts the provision of energy and substances required for the production of nucleic acids and proteins in rapidly multiplying cancer cells [146,147,148]. SIRT4 can enhance the E-cadherin expression and inhibit the expression of N-cadherin and vimentin, thereby suppressing the process of epithelial-mesenchymal transition and reducing the migration and invasive abilities of gastric and colorectal cancer cells [149,150]. In thyroid and gastric cancer cells, overexpression of SIRT4 blocks the progression of the cancer cell cycle by inactivating ERK, p-ERK, cyclin D (ACT/GSK3ß/CyclinD1 pathway) and cyclin E [151,152,153,154]. In addition, SIRT4 can inhibit mTOR and CtBP, and then, suppress glutamine metabolism by inhibiting GDH. Several studies have shown that low levels of SIRT4 lead to an increase in glutamine metabolism and promotes the progression of cancer [155,156]. Interestingly, glutamine plays an important role in the cell cycle during the transition of the cell from the G1 phase to the S. Jeong et al. showed that DNA damage triggers an upregulation of SIRT4 expression, which in turn inhibits glutamine metabolism and halts the cell cycle. This cellular response promotes self-defense and ultimately acts as a tumor suppressor [155]. Tong et al. identified the potential role of SIRT4 in the stimulation of ROS and modulation of apoptosis in Clear cell renal cell carcinomas (ccRCC) through the inhibition of HIF-1α/HO-1 by phosphorylation of p38-MAPK [157] (Table 4 and Table 5).

#### 3.1.5. SIRT5

SIRT5 is still a rather poorly studied sirtuin. At the same time, some research demonstrates its increased expression in some types of oncological diseases (non-small cell lung cancer, breast cancer, Waldenstrom’s macroglobulinemia, hepatocellular carcinoma and colorectal cancer) [158,159,160], whereas other investigations reveal reduced levels of SIRT5 (endometrial carcinoma, head and neck squamous cell carcinoma and hepatocellular carcinoma) [161,162]. Like other sirtuins, the activity of SIRT5 appears to be influenced by the molecular and biochemical conditions of the cancer. Studies suggest that increased expression of SIRT5 can reduce the production of ammonia, leading to a decline in autophagy and mitophagy in MDA-MB-231 and C2C12 human breast cancer cells [163]. Polletta et al. described that ammonia-induced autophagy and mitophagy may play a protective role in cancer cells, making them able to survive chemotherapy or other environmental stresses such as hypoxia or starvation. Lin et al. wrote that SIRT5 desuccinylates and activates SOD1, which leads to a decrease in ROS levels, whereas suppressed SOD1 plays an important role in the growth of lung cancer cells [164]. SIRT5, much like SIRT1, has the ability to facilitate the deacetylation of promyelocytic leukemia tumor suppressor protein in response to H_2_O_2_ exposure. This deacetylation event is critical for the protein to be properly localized within the nucleus and carry out its tumor suppressor function [165]. In their study, Li and colleagues reported that SIRT5 has the ability to counteract metabolic abnormalities and apoptosis resistance in glioma cells harboring IDH1 mutation, resulting in impaired cell growth both in vitro and in vivo [166]. By examining the IDH1 mutation in glioma cells, researchers have discovered that this mutation results in increased succinylation of mitochondrial proteins. This alteration leads to dysfunction in the mitochondria and the accumulation of the anti-apoptotic protein BCL-2. Consequently, the cells become resistant to apoptosis. SIRT5 participates in the inhibition of acyl-CoA oxidase 1 (ACOX1) through desuccinylation [161]. Chronic liver disease and hepatocellular carcinoma are the consequences of ACOX1 activation, which disrupts the redox homeostasis in liver tissues. SIRT5-mediated desuccinylation prevents dimerization of ACOX1 and reduces its activity [161]. Reduced expression of SIRT5 in clear cell renal cell carcinoma may accelerate the Warburg effect due to hypersuccinylation of PDHA1 [167]. A recent study by Choi et al. showed for the first time the relationship between SIRT5 and prostate cancer metastasis. SIRT5-mediated inhibition of the PI3K/AKT/NK-kB pathway decreases with secondary metastasis from bone to other tissues [168].

As a promoter of cancer development, SIRT5 can activate the expression of NRF2 and its related targets, which aid in protecting against oxidative stress and xenobiotics [169]. It negatively regulates SUN2, a tumor suppressor, which in turn reduces the expression of GLUT1 and LDHA and inhibits the Warburg effect [170]. Additionally, SIRT5 modulates the activity of PDC and SDH, which are associated with cancer cell metabolism reprogramming and neoplasia [171]. SIRT5 is involved in folic acid metabolism, which promotes the growth of cancer cells [171]. By binding and succinylating pyruvate kinase M2 (PKM2), SIRT5 inhibits its activity, resulting in the buildup of glycolytic intermediates and promoting cancer growth [172]. Additionally, SIRT5 can stimulate the expression of the transcription factor E2F1, which acts as a suppressor [159]. Du et al. demonstrated that SIRT5 deactivates SDHA, the A subunit of SDH, resulting in the buildup of succinate. This succinate then binds to TrxR2, causing its activation and disruption of redox homeostasis, a crucial factor in the development of chemotherapy resistance [173] (Table 4 and Table 5).

#### 3.1.6. SIRT6

SIRT6 overexpression suppresses tumors in ovarian cancer and glioma cells by inhibiting Notch3 expression [174]. Additionally, it promotes apoptosis by blocking ERK1/2 signal transmission [175], which in turn inhibits MAPK pathway gene expression and prevents proliferation. A negative correlation was found between SIRT6 and PKM2 in contrast to the effect of SIRT5 [176]. SIRT6 is involved in blocking the Warburg effect, similar to SIRT3 and SIRT4. SIRT6 removes the acetyl group from H3K9 on promoters of glycolytic genes and co-represses HIF-1α. This leads to increased expression of glycolytic genes such as LDH, pyruvate dehydrogenase-1 kinase (PDK1), phosphofructokinase-1 (PFK1), and glucose transporter-1 (GLUT1) [177]. The deubiquitination of SIRT6 by USP10 and USP48 inhibits cancer formation by preventing the growth and development of cancer cells. This is achieved through the degradation of c-Myc by p53 and SIRT6, which halts the progression of the cell cycle [178,179]. Moreover, SIRT6 suppresses the JAK2/STAT3 signaling pathway, which typically becomes activated in the progression of various cancers [180]. It also diminishes the levels of NF-κB, leading to an increase in the pro-apoptotic protein Bax and a decrease in the anti-apoptotic factor Bcl-2 [181]. Like SIRT1, SIRT6 participates in the suppression of survivin transcription by deacetylation of H3K9 on its promoter [182]. SIRT6, activating FOXO3a, positively regulates apoptosis, promotes protection from oxidative stress [183]. By decreasing the regulation of the PI3K/Akt/mTOR signaling pathway, SIRT6 is capable of impeding the progression of NSCLC. It is known that SIRT6 acts as a suppressor in hepatocellular carcinoma, lung cancer, nasopharyngeal carcinoma, pancreatic duct adenocarcinoma, melanoma [184,185].

SIRT6 is known as a promoter in prostate cancer, breast cancer, acute myeloid leukemia, head and neck squamous cell carcinomas [184,186,187,188,189]. SIRT6 has been shown to have multiple effects on cell survival and cancer progression. It can deacetylate Ku70, thereby blocking the Bax-mediated internal pathway of apoptosis [190]. Additionally, SIRT6 can deacetylate the anti-apoptotic factor AKT, which in turn phosphorylates the X-linked inhibitor of apoptosis protein (XIAP) [187]. SIRT6 also promotes the inhibition of the expression of tumor suppressors p53 and FOXO3a [191] and participates in the suppression of AMP-activated protein kinase (AMPK) signaling, which ultimately leads to the survival and proliferation of cancer cells [192]. Furthermore, SIRT6 inhibits the IGF/AKT pathway, which is responsible for suppressing autophagy [193], while activating the PI3K/AKT/mTOR pathway, which promotes cancer progression [189]. Finally, SIRT6 regulates the transmission of Wnt/β-catenin signals, which contribute to the progression of cancer [194] (Table 4 and Table 5).

#### 3.1.7. SIRT7

The study of the oncogenic role of SIRT7 has only gained significant attention in the past 5 years. SIRT7, similar to other sirtuins, has been demonstrated to deacetylate and suppress p53 [195]. However, at the same time, Wang et al. showed that retinoid-IFN-induced mortality-19 (GRIM-19) suppresses the development of colorectal cancer through posttranslational regulation of p53. SIRT7 is activated by GRIM-19 and triggers MDM2 ubiquitination, ultimately stabilizing the p53 protein [196]. SIRT7 promotes the growth of gastric cancer cells by inhibiting the expression of the tumor suppressor miR-34a [197]. In breast cancer, SIRT7 mRNA was found to be activated in cancers at early stages, but not at late stages [78]. Resveratrol treatment activates the oncosuppressive properties of SIRT7, which is evidenced by its ability to deacetylate SMAD4 on K428, leading to destabilization and disruption of TFR-β signaling and epithelial mesenchymal junction (EMT) in vitro. In mice, resveratrol treatment was found to decrease EMT and lung metastases by destabilizing SMAD4, implicating SIRT7 in resveratrol’s anticancer effects [198]. Tang et al. identified the GSK3ß-SIRT7 axis, which provides enhanced anticancer effect during chemotherapy [199]. When AMPK phosphorylates SIRT7, it leads to the subsequent phosphorylation of glycogen synthase kinase 3β (GSK3β). This phosphorylation stabilizes SIRT7 by dissociating it from the E3 ligase UBR5 [199]. Overall, high levels of SIRT7 have been found in stomach cancer, prostate adenocarcinoma, skin, liver, lung, and breast cancers [200] (Table 4 and Table 5).

SIRT1-7 have the ability to function as both oncosuppressors and oncopromoters, contingent upon the particular type of cancer and the intracellular signaling pathways involved. However, due to conflicting reports on the role of sirtuins in the development of cancer, there is a clear necessity for a more thorough examination of their functional activities. As they have the potential to be targeted for the creation of novel therapeutic options for a variety of malignant neoplasms, it is imperative that their functional activity is studied in greater detail.

### 3.2. PARPs

PARPs are enzymes that transfer ADP-ribose groups to target proteins, regulating various nuclear and cytoplasmic processes that ensure the survival or death of cells (modulation of chromatin structure, transcription, replication, DNA repair, etc.) [35,79,201,202,203]. PARP consists of four domains that serve different functions: a DNA-binding domain, a caspase-cleaved domain, an auto-modification domain, and a catalytic domain. The DNA-binding domain comprises two zinc finger motifs, and its primary role is to bind to damaged DNA, causing a change in the protein’s shape. This binding happens independently of the other domains and is critical in a programmed cell death model that inhibits caspase cleavage of PARP. The auto-modification domain’s function is to release the protein from the DNA after catalysis and also plays a crucial role in cleavage-induced inactivation. When a single-strand break is detected, PARP binds to the DNA, goes through a structural change, and starts synthesizing a chain of polymeric adenosine diphosphate ribose (poly (ADP-ribose) or PAR). This PAR chain acts as a signal for other DNA-repairing enzymes like DNA ligase III, DNA polymerase beta, and scaffolding proteins like X-ray cross-complementing gene 1 (XRCC1). Once the repair is complete, Poly(ADP-ribose) glycohydrolase (PARG) degrades the PAR chains. Moreover, caspase-3,7 cleavage is responsible for the deactivation of PARP. Typically, this deactivation process occurs when there is extensive DNA damage in a system. In such cases, the amount of energy required to repair the damage is impractical, and as a result, programmed cell death is initiated to conserve energy for other cells in the tissue. In addition to degradation, recent studies have shown that PARP can also be downregulated through reversible mechanisms, including an “autoregulatory loop” [204]. PARP1 itself drives this loop, which is modulated by the YY1 transcription factor.

To date, 17 different genes encoding all members of the PARP family are known [205]. A brief description of them is given in Table 6. However, PARP-1, 2 are the most studied [206]. PARP, similar to sirtuins, employs NAD^+^ as its primary substrate for reactions and produces nicotinamide and poly(ADP-ribose) [207,208]. In this regard, the functionally competitive interaction between PARPs and SIRTs, mediated by the NAD^+^ level, can determine the future fate of the cell. Excessive activation of a specific enzyme results in reduced activity of other enzymes. In particular, heightened activation of PARP-1 causes a swift decline in the levels of NAD^+^ within cells. As a result, the Salvage pathway is initiated to restore NAD^+^ levels, but this process also reduces intracellular ATP levels. Consequently, apoptosis-inducing factors (AIF) are released, leading to reduced cellular energy and eventual cell death [12,209,210,211]. PARP activation can cause a bioenergetic crisis that ultimately results in necrotic cell death, often accompanied by inflammation [209]. On the other hand, SIRT1 plays a role in reducing PARP-1 activity by deacetylation. The careful control of PARP activity, using NAD^+^ and SIRT levels, is crucial in preventing various diseases, including cancer. It is worth noting that PARP-1 responds more quickly to oxidative stress than sirtuins, due to its stronger affinity for and faster binding kinetics to NAD^+^ [212]. It is also curious that the PARP inhibition stimulates an increase in the activity of not all sirtuins at once, but only SIRT1, not SIRT2 or SIRT3. Thus, subcellular localization of enzymes plays an important role [11]. This fact indicates the existence of independently regulated NAD^+^ pools in the cell in its different compartments [11].

In this regard, suggestions have been made that PARP inhibition may be one of the means of indirect regulation of SIRT1 activity through an increase in NAD^+^ levels [227,228]. However, blocking PARP jeopardizes the protection of cells from genotoxic damage similar to NAMPT inhibitors. Thus, therapy, which additionally includes long-term exposure to healthy tissues, is a strategy with high risks of serious side effects.

Nevertheless, today, such PARP inhibitors as olaparib, rukaparib, niraparib and talazoparib, approved by the FDA, are successfully used and European Medical Agency (EMA) in the treatment of ovarian cancer, pancreatic cancer, prostate cancer in inpatient patients with BRCA1/BRCA2 mutations, as well as independently of them [229]. In addition, these drugs are actively undergoing clinical trials for the development of other oncological diseases: non-small cell lung cancer and stomach cancer. Olaparib (2009) is used in the treatment of breast cancer, its metastasis and pancreatic cancer [230,231]. Olaparib is the safest compared to conventional chemotherapeutic drugs, as it has demonstrated mild and reversible side effects during clinical trials [232]. In 2016, rukaparib was approved for the treatment of adult ovarian cancer patients with germinal and/or somatic BRCA mutation [233]. Niraparib was granted approval in 2017, and it offered extended progression-free survival for patients with BRCA1/2 mutant tumors compared to olaparib. However, the use of this medication also led to the emergence of severe side effects, including anemia, neutropenia, and thrombocytopenia [234]. According to clinical trials [235], Talazoparib is a PARP inhibitor that is still in its early stages of development. The mechanism of action of all PARP inhibitors is to compete with NAD^+^ for the catalytic pocket of PARP [236]. By attaching to the catalytic site, PARP1 inhibitors create hydrogen bonds with Gly, Ser, and Glu, as well as hydrophobic stacking interactions with two Tyr residues inside the nicotinamide-binding pocket [237].

PARPs, much like sirtuins, have the ability to directly impact the development of cancer. Researching not only the mechanisms of individual enzyme groups in the formation of malignant processes within a cell, but also their interactions with each other, may serve as the foundation for developing more effective therapeutic approaches to treating oncological pathologies.

## 4. Modulation of the Ratio of NAD^+^, SIRTs and PARPs as a Pharmacological Effect on Cancer

**Figure 3:**
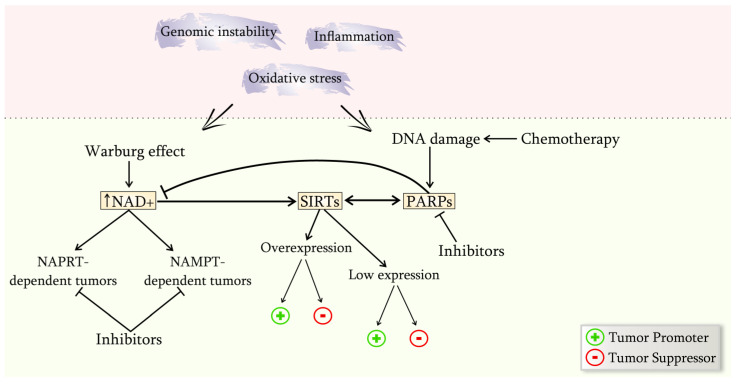
Ways of NAD^+^/SIRTs/PARPs interactions in the conditions of the cancer process development. A cancer cell exhibits genomic instability and inflammation, resulting in DNA damage accumulation and Warburg effect formation. The shift in the NAD^+^/NADH balance during the development of the Warburg effect impacts the functioning of other essential cellular structures. Sirtuins respond to elevated NAD^+^ levels by altering their activity, influencing PARP function. However, conflicting literature reports indicate that sirtuin overexpression and downregulation both occur in cancer cells, leaving uncertainty about their role in tumor growth. Additionally, PARPs are activated by DNA damage, leading to a decrease in NAD^+^ levels and potentially inducing energy stress and cell death, which is detrimental to cancer cells. Interestingly, SIRT1 can translocate between the nucleus and cytoplasm, with cytoplasmic localization in tumor cells increasing cell sensitivity to apoptosis [7,238,239]. This suggests that SIRT6 and SIRT7 may play a greater role in the early stages of cancer development when interacting with PARPs, and SIRT1 may move to the nucleus depending on the signaling pathways predominant in each tumor type. Consequently, reprogrammed signaling pathways aim to prevent the activation of apoptosis or necrosis by active PARP function.

One crucial aspect of maintaining the viability and malignancy of a cancer cell is the reprogramming of its energy-biochemical metabolism. The Warburg effect, alterations in SIRTs’ functional activity across different cell compartments, and their interaction with PARPs are critical factors in cancer development. It is essential to note that the molecular and biochemical changes that occur within the cell heavily rely on the cancer stage, neoplasm type, and the processes or events that triggered cancer formation, as evidenced by extensive literature. By studying the biochemical reprogramming of cancer cells in combination, it is possible to discover innovative approaches to cancer therapy that consider the functioning of multiple signaling pathways and their interplay (Figure 3).

NAD^+^ cofactors (e.g., nicotinamide riboside) can be an effective and safe candidate aimed at normalizing the ratio of NAD^+^, SIRTs and PARPs in the cells. Numerous studies have shown that nicotinamide riboside has more advantageous kinetic properties compared to its analogues. It’s crucial to recognize that the NAD^+^/SIRTs/PARPs interaction signals have a specific purpose: to hinder the progression of oncogenesis. This can be accomplished by halting the cell cycle and initiating repair mechanisms, or if repair efforts prove unsuccessful, by initiating apoptosis. Nicotinamide riboside, normalizing NAD^+^ levels in cell compartments, is able to affect the SIRTs activity, triggering a number of molecular mechanisms that prevent the carcinogenesis development (SIRT/Notch, SIRT/FOXO1, AMPK/SIRT1/NF-κB, SIRT/VEGF, SIRT/CTGF, SIRT/ATP, SIRT/p53, SIRT/ROS/HIF-1α, SIRT/ACT/GSK3β/CyclinD1, SIRT/mTOR, SIRT/CtBP, SIRT/SMAD), and on the PARPs activity stimulating the development of apoptotic processes. However, these molecular and biochemical aspects have yet to be studied.

The function of sirtuins is ultimately targeted by all known inhibitors of either NAD^+^ or PARPs. NAD^+^ depletion suppresses SIRTs, leading to cancer regression. On the other hand, inhibiting PARPs activates SIRTs by increasing NAD^+^, which also hinders cancer progression. However, at the same time, all drugs have a high toxic effect on the surrounding healthy tissues. There is a question of delivery, so as not to harm normally functioning cells. Nicotinamide riboside, which is a natural precursor to NAD^+^ [240], can safely and directly impact the function of both sirtuins and PARPs.

Therefore, given the role of the interaction between SIRTs and PARPs in the development of cancer, it is reasonable to view the manipulation of this interaction as a valid means of generating an anti-tumor effect. To this end, the co-factor NAD^+^ may be employed as a modulator.

## Figures and Tables

**Table 1:** Molecular pathways regulating NAMPT expression in cancer.

NAMPT-Regulating Pathways	Interaction of Essential Factors	References
c-MYC/SIRT1	c-MYC → NAMPT → ↑NAD^+^ level → SIRT1 activation → ↑c-MYC transcriptional activity	[41,42,43]
HMGA1/NAD^+^	HMGA1 → NF-κB activity → NAMPT expression → ↑NAD^+^	[44]
FOXO1	FOXO1 → ↓NAMPT expression	[45]
microRNA	miR-381, miR-206, miR-494, miR-154, miR23b, miR26b → ↓NAMPT expression	[46,47,48,49,50,51]
SIRT6/SIRT1	SIRT6/SIRT1 → ↑NADPH	[52]
C/EBPβ	C/EBPβ → ↑NAMPT expression	[53]

↑—enhance the activity; ↓—suppress the activity.

**Table 3:** Cellular distribution and primary functions of the seven classes of sirtuins (SIRTs).

SIRT Classes	Cellular Compartment	Dominant Functions
SIRT1	Nucleus/Cytoplasm	Cell cycle regulation, cell proliferation, gene expression, chromatin modification, genomic stability, energy metabolism, stress response, cell survival
SIRT2	Nucleus/Cytoplasm	Cell cycle regulation, cell survival
SIRT3	Mitochondrion	Energy metabolism, stress response, cell survival
SIRT4	Mitochondrion	Energy metabolism, cell cycle regulation, stress response, cell survival
SIRT5	Mitochondrion	Energy metabolism, stress response, cell survival
SIRT6	Nucleus	Energy metabolism, stress response, cell survival
SIRT7	Nucleus	Energy metabolism, stress response, cell survival, genomic stability

**Table 4:** The involvement of molecular factors in the stimulating effect of sirtuins on the growth of cancer tissue.

Class of Regulatory Sirtuins	Molecular Factors	Changes in the Functioning of Molecular Markers
SIRT1, SIRT2, SIRT3, SIRT4, SIRT5, SIRT6, SIRT7	NAD^+^	?
SIRT1, SIRT2, SIRT6, SIRT7	p53	Non-functional
SIRT1, SIRT5, SIRT6	E2F1	Inhibition
SIRT1	HIC1	Inhibition
SIRT1, SIRT3, SIRT6	c-MYC	Activation
SIRT1, SIRT3, SIRT6	KU70	Activation
SIRT2	α-tubulin	Activation
SIRT1, SIRT2, SIRT6	β-catenin	Activation
SIRT2	PEPCK1 (RAS/ERK/JNK/MMP-9)	Activation
SIRT5	SUN2	Inhibition
SIRT5	NRF2	Activation
SIRT5	PKM2	Inhibition
SIRT5	SDHA	Inhibition
SIRT1	miR-22, miR-93, miR-217, miR-449	Inhibition
SIRT2	miR-21, miR-138	Inhibition
SIRT3	miR-224	Inhibition
SIRT1, SIRT6, SIRT7	mir-34a	Inhibition
SIRT6	mir-122	Inhibition

HIC1, hypermethylated in cancer 1 protein; NRF2, nuclear factor erythroid 2-related factor 2; PEPCK1, phosphoenolpyruvate carboxykinase 1; PKM2, pyruvate kinase isozymes M2; SDHA, succinate dehydrogenase complex, subunit A; ?—no exact information.

**Table 5:** The involvement of molecular factors in the inhibitory effect of sirtuns on the growth of cancer tissue.

Class of Regulatory Sirtuins	Molecular Factors	Changes in the Functioning of Molecular Markers
SIRT1, SIRT2, SIRT3, SIRT4, SIRT5, SIRT6, SIRT7	NAD^+^	?
SIRT1, SIRT2, SIRT6, SIRT7	p53	Functioning
SIRT1, SIRT6, SIRT7	MDM2	Inhibition
SIRT1, SIRT2, SIRT3, SIRT6	FOXO	Inhibition
SIRT1, SIRT6	Notch	Inhibition
SIRT1, SIRT2	VEGF/CTGF/ACLY	Inhibition
SIRT1, SIRT3, SIRT4, SIRT6	HIF-1α	Inhibition
SIRT1, SIRT3, SIRT6, SIRT7	AMPK	Activation
SIRT1, SIRT2, SIRT6	NF-κB	Inhibition
SIRT2, SIRT3, SIRT4	p38-MAPK/ERK	Inhibition
SIRT4	N-cadherin/vimentin	Inhibition
SIRT4	E-cadherin	Activation
SIRT4	CyclinE	Inhibition
SIRT4, SIRT7	AKT/GSK3β/CyclinD	Inhibition
SIRT5	PI3K/AKT/NF-κB	Inhibition
SIRT6	PI3K/AKT/mTOR	Inhibition
SIRT1	BRCA1	Deficiency
SIRT2	APC/C	Inhibition
SIRT3	HMG-CoA synthase	Activation
SIRT3, SIRT4	GDH	Inhibition
SIRT3	Akt/PKB	Inhibition
SIRT3	IRP1	Inhibition
SIRT2, SIRT3	Skp2	Inhibition
SIRT5	ACOX1	Inhibition
SIRT5	SOD1	Activation
SIRT7	SMAD4	Inhibition
SIRT6	JAK2/STAT3	Inhibition

ACLY, ATP citrate synthase; ACOX1, peroxisomal acyl-coenzyme A oxidase 1; AKT, protein kinase B; AMPK, 5′ AMP-activated protein kinase; APC/C, anaphase-promoting complex; GDH, glutamate dehydrogenase; BRCA1, breast cancer type 1 susceptibility protein; CTGF, connective tissue growth factor; FOXO, forkhead box O; GSK3β, glycogen synthase kinase-3 beta; HIF-1α, hypoxia-inducible factor-1α; IRP1, iron-responsive element-binding proteins; JAK2/STAT3, janus kinase/signal transducer and activator of transcription; MDM2, mouse double minute 2 homolog; NF-κB, nuclear factor kappa-light-chain-enhancer of activated B cells; p38-MAPK/ERK, mitogen-activated protein kinase/extracellular signal-regulated kinases; PI3K, phosphoinositide 3-kinases; Skp2, S-phase kinase-associated protein 2; SOD1, superoxide dismutase; VEGF, vascular endothelial growth factor; ?—no exact information.

**Table 6:** Characterization of the PARP family based on their functional and structural distinctions.

Type of PARPs	Functional and Structural Distinctions	Participation in the Growth of Cancer Tissue
PARP1, PARP2, PARP3	DNA-dependent	Overexpressed [213,214]
PARP7, PARP12, PARP13.1, PARP13.2	Cys-Cys-Cys-His zinc finger	Overexpressed [215,216]
PARP9, PARP14, PARP15	Macro-PARPs	Overexpressed [217,218]
Tankyrase 1 (PARP-5a), tankyrase 2 (PARP-5b)	Tankyrases	Overexpressed [219]
PARP4	Lacks an N-terminal DNA binding domain (activated by protein-protein interaction)	Cancer susceptibility gene [220,221]
PARP6	Less studied or not typical	Acts as an oncogene [222]; Acts as a tumor suppressor [223]
PARP8	Less studied or not typical	-
PARP10	Less studied or not typical	Overexpressed [224]
PARP11	Less studied or not typical	Participates in the disorders of antitumor immunity [225]
PARP16	Less studied or not typical	Acts as an oncogene [218,226]

## Data Availability

Not applicable.
